# Impact of the COVID-19 Pandemic on Diagnosis and Management of Gynecological Cancer: A Single-Center Analysis

**DOI:** 10.3390/medicina58121862

**Published:** 2022-12-16

**Authors:** Dora Antunes, Lisandra Mendonça, Ângela Melo, Sónia Gonçalves, Francisco Nogueira Martins, Nuno Nogueira Martins

**Affiliations:** 1 Department of Gynecology, Centro Hospitalar e Universitário de Coimbra, Praceta Prof. Mota Pinto, 3004-561 Coimbra, Portugal; 2 Faculty of Medicine, University of Coimbra, Azinhaga de Santa Comba, Celas, 3000-548 Coimbra, Portugal; 3 Department of Gynecology, Centro Hospitalar Tondela-Viseu, Avenida Rei Dom Duarte, 3504-509 Viseu, Portugal

**Keywords:** COVID-19 pandemic, gynecologic neoplasms, diagnosis, cancer staging, treatment, gynecologic surgery, chemotherapy, radiotherapy

## Abstract

*Background and Objectives*: The COVID-19 pandemic impacted health systems worldwide, particularly cancer care. Because the actual implications of these changes on gynecological oncology healthcare are still unclear, we aim to evaluate the impact of this pandemic on the diagnosis and management of gynecological cancer. *Materials and Methods*: This is a single-center retrospective observational study, including patients diagnosed with gynecological malignancies between January 2019 and December 2021. Patients were included into three groups based on the timing of cancer diagnosis: pre-pandemic (2019), pandemic with high restrictions (2020) and pandemic recovery (2021). *Results*: Overall, 234 patients were diagnosed with gynecological cancer during the period of study. A decrease in the number of newly diagnosed cervical cancers and other rare tumors (leiomyosarcoma, invasive hydatidiform mole) was apparent in 2020. Some aggressive histological types of endometrial and ovarian cancer were more commonly diagnosed in the pandemic recovery group (*p* < 0.05), although no differences were demonstrated concerning tumor staging in all gynecological cancers. The median time between the first multidisciplinary team meeting and the treatment initiation was higher after the COVID-19 pandemic in endometrial cancer (23.0 vs. 34.0 vs. 36.0 days, *p* < 0.05). Patients with ovarian cancer were more frequently proposed for neoadjuvant therapy in 2020 compared to the other periods (33.3% vs. 55.0% vs. 10.0% *p* < 0.05). A significant reduction in the laparoscopic approach was observed during 2020 in endometrial cancer (32.1% vs. 14.3% vs. 36.4%, *p* < 0.05). No significant differences were registered regarding median hospitalization days or intra- and post-operative complications between these periods. *Conclusions*: The COVID-19 pandemic had a significant impact on the diagnosis and management of most gynecological malignancies, namely, on time to first treatment, chosen oncological therapies and surgical approaches. These results suggest important clinical and healthcare implications that should be addressed in future prospective studies.

## 1. Introduction

On 11 March 2020, the World Health Organization declared COVID-19 a pandemic on the basis of its spread and severity [1], which has led to a global crisis with a huge impact worldwide and a disruption of most health systems and economies. Cancer care was among the most impacted areas across the whole spectrum of prevention, diagnosis, surgery, oncology treatments and palliative care [2,3,4].

The COVID-19 pandemic resulted in a significant reconfiguration of health services and care pathways in order to protect vulnerable patients from infection whilst providing care with limited resources and staffing. Due to immunosuppression that arises from cancer and its treatments, as well as other overlapping risk factors, cancer patients are considered a vulnerable population [4,5]. A Chinese study published in February 2020 described a 3.5 times higher risk of needing mechanical ventilation, intensive care admission or dying in patients with a history of cancer than in those without this precedent [6]. Additionally, the CovidSurg project, an international multicenter study including 1128 patients undergoing surgery, reported 30-day mortality rates of 19% in elective surgeries, 26% in emergency surgeries and 27% in cancer surgeries when patients were infected by the severe acute respiratory syndrome coronavirus 2 (SARS-CoV2) in the perioperative period [7].

Attending to these facts, numerous guidelines, statements and expert opinions have been published on the management of gynecological cancer during the COVID-19 pandemic. Indeed, the international gynecologic cancer community developed modifications to clinical care concerning cancer treatments and their timelines from first presentation to relapse and palliation [8]. The reallocation of resources and adaptation of gynecologic cancer services imposed some adjustments such as remote consultations, reduced hospital visits, routine COVID-19 testing, reduced elective surgeries and use of COVID-19-free surgical areas for the delivery of complex cancer care, with priority levels for cancer treatments established to guide decision-making by multidisciplinary teams [2,3,4,8,9,10].

Despite the pragmatic modifications that have been globally implemented among services, their real impact on gynecological oncology healthcare is still unclear. A Cancer Research United Kingdom (CRUK) study reported a substantial impact of the COVID-19 pandemic on cancer diagnosis, with more than 350,000 fewer people than usual being referred for suspected cancer between March and September 2020, largely owing to fewer people seeking primary care advice [11]. Moreover, a global modeling analysis suggested that around 38.0% of cancer surgeries and 82.0% of benign surgical procedures would have been canceled or postponed during the 12 weeks of peak disruption due to the COVID-19 pandemic [12]. An international patient survey conducted by the European Society of Gynecological Oncology (ESGO)-European Network of Gynecological Cancer Advocacy Groups (ENGAGe) also demonstrated that patients were more fearful of changes to planned oncological treatments and possible cancer progression (70.9%) than developing COVID-19 [13].

Moreover, although few studies have been published on the impact of the COVID-19 pandemic on gynecological cancer healthcare, most of them have been based on small sample sizes and have been restricted to the first wave of SARS-CoV2 infection, without continuous feedback relative to the subsequent periods [14,15,16,17,18]. Hence, there is an emergent need to properly understand the impact of the COVID-19 pandemic on the care of gynecologic cancer patients.

As in other countries of the world, significant restrictions affecting healthcare occurred in Portugal after March 2020, with a progressive recovery in 2021. Therefore, this study aims to assess the impact of the COVID-19 pandemic on the diagnosis and management of gynecological cancer, evaluating the pre-pandemic, pandemic with high restrictions and pandemic recovery periods.

## 2. Materials and Methods

### 2.1. Study Design

This is a retrospective observational study conducted at the Gynecology Department of Centro Hospitalar Tondela-Viseu, Portugal. We included all patients diagnosed with gynecological cancer (endometrium, ovary, fallopian tube or peritoneum, cervix, vagina and vulva) that were referred to our department between 1 January 2019 and 31 December 2021. Patient medical records were reviewed after obtaining informed consent, and data were collected regarding age, postmenopausal status, tumor characteristics (histology, staging), multidisciplinary team meetings (MDTM), oncological treatments (neoadjuvant, surgical, adjuvant and/or palliative), days of hospitalization and intra- and post-operative complications. Patients were included into three groups based on the timing of cancer diagnosis: pre-pandemic (2019), pandemic with high restrictions (2020) and pandemic recovery (2021). All investigations included a guarantee of anonymity and were conducted according to the principles expressed in the Declaration of Helsinki.

### 2.2. Statistical Analysis

Descriptive and inferential analysis was performed using SPSS Statistics software version 26.0 (IBM Corp., Armonk, NY, USA). Continuous variables were expressed as median and interquartile ranges (IQR) given their non-normal distribution, and categorical variables were presented as absolute numbers and percentages. The Kruskal–Wallis test and chi-squared test were used for group comparisons among continuous and categorical variables, respectively. A two-sided *p*-value <0.05 was considered statistically significant.

## 3. Results

**Figure 1:**
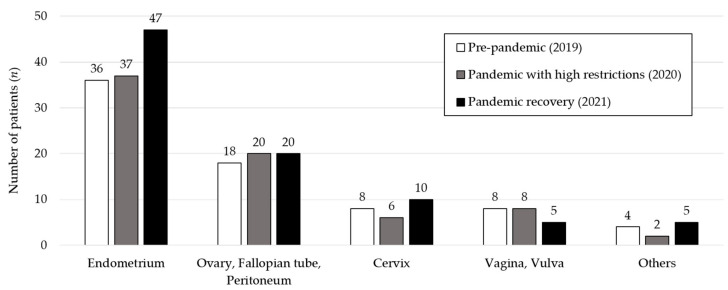
Number of patients diagnosed with gynecological cancer according to its location and pandemic period. Others: leiomyosarcoma, invasive hydatidiform mole, synchronous endometrial and ovarian carcinoma.

Overall, 234 patients were diagnosed with gynecological cancer and referred to our department between 2019 and 2021. The patients’ distribution according to cancer location and timing of diagnosis is shown in Figure 1.

### 3.1. Endometrial Cancer

A total of 120 patients were diagnosed with endometrial cancer between 2019 and 2021, of which 36 were detected in the pre-pandemic period (2019), 37 in the pandemic with high restrictions period (2020) and 47 in the pandemic recovery period (2021).

Patients diagnosed before the COVID-19 pandemic were older than those diagnosed after that period (*p* < 0.05). On the other hand, there was no statistically significant difference regarding postmenopausal status.

Concerning tumor characterization, the histological types significantly differed between groups, with a higher number of cases of undifferentiated (6.4%) and mixed (8.5%) types in the pandemic recovery period compared to the other periods (*p* = 0.001). Nonetheless, no statistical differences were reported considering tumor staging.

The median time between the first MDTM and the treatment initiation was higher after the COVID-19 pandemic (34.0 days in 2020 and 36.0 days in 2021) in comparison to the pre-pandemic period (23.0 days) (*p* < 0.05).

Regarding surgical management, the laparoscopic approach was significantly reduced during the pandemic with high restrictions (*p* < 0.05), and a significant increase in sentinel lymph node biopsies was observed in 2020 and 2021 (*p* < 0.001). The median time between surgery and histological assessment was also lower after the COVID-19 pandemic (*p* < 0.05). There were no differences regarding hospitalization days, intra- and post-operative complications and time between surgery and adjuvant therapy.

Epidemiological, clinical and pathological patterns of the patients diagnosed with endometrial cancer are presented in Table 1.

### 3.2. Ovarian, Fallopian Tube and Primary Peritoneal Cancer

The epidemiological, clinical and pathological characterization of patients with ovarian, fallopian tube and primary peritoneal cancer are described in Table 2. Among the 58 cases diagnosed between 2019 and 2021, 18 occurred in the pre-pandemic period (2019), 20 during the pandemic with high restrictions period (2020) and 20 in the pandemic recovery period (2021).

There were no statistical differences regarding patient age at diagnosis and postmenopausal status. Considering tumor histological type, a higher number of cases of borderline ovarian tumor (45.0%), clear cell carcinoma (10.0%) and carcinosarcoma (5.0%) was observed in the pandemic recovery period compared to the other periods (*p* < 0.05), though no differences were registered regarding tumor staging.

The percentage of patients submitted to neoadjuvant therapy was higher in the pandemic with high restrictions period (55.0%) in comparison to the pre-pandemic (33.3%) and pandemic recovery (10.0%) periods (*p* < 0.05). Time between the first MDTM and treatment initiation, surgical approach, hospitalization days, intra- and post-operative complications and time between surgery and histological assessment did not differ between groups. The median days between surgery and adjuvant therapy was higher in the pandemic recovery period than in the other periods (*p* < 0.05).

### 3.3. Cervical Cancer

Twenty-four patients were diagnosed with cervical cancer during the period of study (8 in the pre-pandemic, 6 in the pandemic with high restrictions and 10 in the pandemic recovery).

Regarding the patients’ demographics, although the median age at diagnosis was lower in the pandemic recovery group (47.5 years) than in the pre-pandemic (63.5 years) and the pandemic with high restrictions (69.0 years) groups, this difference is not statistically significant. Nevertheless, there were fewer postmenopausal patients with cervical cancer in 2021 (40.0%) compared to 2019 (75.0%) and 2020 (100.0%) (*p* < 0.05). There were no statistical differences between groups considering tumor characteristics (histological type and tumor staging).

With respect to treatment management, no differences were observed concerning the time between the first MDTM and treatment initiation, type of treatment, hospitalization days and time between surgery and histological assessment. No intra- or post-operative complications were reported during this period.

The characterization of patients diagnosed with cervical cancer is summarized in Table 3.

### 3.4. Vaginal and Vulvar Cancer

All the epidemiological, clinical and pathological data of patients diagnosed with vaginal and vulvar cancer are reported in Table 4.

Among the 21 patients with the diagnosis of vaginal or vulvar cancer between 2019 and 2021, 8 were diagnosed in the pre-pandemic period (2019), 8 in the pandemic with high restrictions period (2020) and 5 in the pandemic recovery period (2021).

The median age at diagnosis did not significantly differ between groups. Nevertheless, the percentage of women in postmenopause was lower in 2021 (60.0%) compared to 2019 (100.0%) and 2020 (100.0%) (*p* < 0.05).

Considering the tumor characterization, no statistically significant differences were observed in histological type or tumor staging, despite the higher percentage of advanced tumors diagnosed in the pandemic recovery period (60.0%) in comparison with pre-pandemic (0.0%) and pandemic with high restrictions (37.5%) periods.

Although not statistically significant, the median time between the first MDTM and treatment initiation was higher in the pandemic recovery group (49.0 days versus 27.0 days in the other groups). The same was observed regarding the type of treatment, with more patients submitted to chemoradiotherapy in the pandemic recovery group (40.0%) and more patients selected for palliative approaches in the pandemic with high restrictions group (12.5%). There were no other significant differences between groups considering the time between surgery and histological assessment, time of hospitalization and intra- and post-operative complications.

### 3.5. Other Types of Cancer

Other types of gynecological cancer were also diagnosed during the period of study. In the pre-pandemic group (2019), two patients presented with leiomyosarcomas, one had an invasive hydatidiform mole and the other was diagnosed with a synchronous endometrial and ovarian carcinoma. In the pandemic with high restrictions group (2020), one patient presented with leiomyosarcoma and the other had an invasive hydatidiform mole. In the pandemic recovery group (2021), five patients were referred to our department due to leiomyosarcomas.

## 4. Discussion

To our knowledge, this is the first study in Portugal reporting the impact of the COVID-19 pandemic on the diagnosis and management of gynecological cancer and one of the first studies worldwide to perform a continuous evaluation of these outcomes comparing the pre-pandemic period to either the pandemic with high restrictions or the pandemic recovery periods.

In the initial phase of SARS-CoV2 infection, prioritization frameworks were issued by international gynecologic oncology societies attempting to balance the risks of treatment in COVID-19-exposed environments with existing limited resources and the possible effect of such delays on oncological outcomes [4,8]. However, the real impact of these adaptations on gynecological oncology healthcare is still poorly understood, and there are some variable findings reported in the literature.

Concerning cancer diagnosis, Bruce et al. [23] suggested that the number of referrals to gynecologic oncology decreased during the early stages of the pandemic, while in a Dutch population-based cohort study [24], the ovarian, vulvar and endometrial cancer volumes remained stable, with a 17.2% decrease in the surgical volume for cervical cancer in 2020 compared to the precedent years. In our study, the diagnosis of cervical and other rare tumors like leiomyosarcomas and invasive hydatidiform moles appeared to decrease in the pandemic with high restrictions period (2020) compared to the other periods. Conversely, endometrial, ovarian, fallopian tube and peritoneum, as well as vaginal and vulvar cancers did not seem to be affected. These findings could be explained by the patients’ fear and concern about SARS-CoV2 infection, the limited access to primary care services during the COVID-19 pandemic and the interruption of the cervical screening program that mitigated and postponed the number of newly diagnosed cervical cancers, particularly in younger women. Additionally, in endometrial cancer, the early occurrence of abnormal uterine bleeding may have led to the patients’ awareness and prompt demand for medical counseling, thus not impairing cancer diagnosis.

Although a few differences are reported in our study regarding tumor histology in endometrial and ovarian cancers, with some aggressive tumors being more frequently diagnosed in the pandemic recovery group, the tumor staging did not significantly differ between groups. In a previous report [24], similar patient and tumor characteristics were demonstrated for cervical, endometrial, ovarian and vulvar cancer during the pre- and post-pandemic periods. Nevertheless, a higher rate of advanced-stage cervical and ovarian cancers was registered during the pandemic period (2020).

Some disparities are demonstrated in our study considering gynecologic cancer management. In endometrial cancer, the median time between the first MDTM and the treatment initiation was statistically higher after the COVID-19 pandemic (34.0 days in 2020 and 36.0 days in 2021) in comparison to the pre-pandemic period (23.0 days). Otherwise, in vulvar cancer, this interval seemed to be higher in the pandemic recovery group (49.0 days versus 27.0 days in the other groups), although this result is not statistically significant. In the other cancers, no significant differences were reported on this issue. These contradictory results were also shown in previous reports, as Bruce et al. [23] stated that the time to evaluation and treatment initiation was not affected, while other retrospective cohort studies described a delay in consultations and cancer treatments [18,25,26,27]. This could be related to the adjustments in gynecologic cancer services, the availability of resources and staffing in a multidisciplinary setting and the status of SARS-CoV2 infection in healthcare services.

The COVID-19 pandemic has been associated with major changes in cancer treatments worldwide. Consistent with previous reports [24], in our study, the percentage of patients with ovarian cancer proposed for neoadjuvant therapy was statistically higher in the pandemic with high restrictions period (55.0%) compared to the other periods (33.3% and 10.0%). This is in accordance with the initial guidelines issued by international gynecologic oncology societies that recommended neoadjuvant therapy instead of primary cytoreductive or interval debulking surgeries in advanced ovarian cancer in order to avoid inpatient hospitalization, surgical morbidity and resource requirements, namely, intensive care needs for patients submitted to complex cancer surgery [4,8,28,29,30].

In the same way, many statements and expert opinions have been published regarding the surgical management of gynecological cancer. One of the most impactful measures was the implementation of priority levels for treatment selection in order to reduce hospital admissions and elective surgeries. This led to significant treatment modifications, particularly to the delay, change and cancelation of surgical plans, or even disruption to usual first-line surgical approaches [14,31,32]. Moreover, in cases of more advanced or relapsed disease, in which treatment is intended to be more life-prolonging and not curative, international guidelines recommended that surgery should be delayed or replaced by systemic or palliative options that could be associated with poorer and less favorable outcomes [32]. These facts were documented by a multicenter prospective cohort study including 3973 cancer patients, in which 1 in 5 patients with canceled surgeries had disease progression, and 1 in 20 died within 3 months after the multidisciplinary decisions [32].

Considering the surgical route, some concerns about laparoscopy were also initially stated, especially in surgeries that involve the gastrointestinal tract, as this approach is considered an aerosol-generating procedure, thus being associated with an increased risk of SARS-CoV2 transmission. However, the available evidence does not confirm this theory, and minimally invasive surgery continued to be recommended during the pandemic, maintaining the standard surgical approaches [4,28,33,34,35,36]. Hence, in our study, a significant reduction in the laparoscopic route was observed during the pandemic with high restrictions period in patients with endometrial cancer, although no differences were detected in the other gynecological cancers.

Despite the opposing data that were published in the beginning of the pandemic, with some studies reporting a significantly higher rate of postoperative complications and 30-day mortality [15] and others demonstrating similar major morbidity and mortality from gynecologic cancer surgery during the pandemic and in pre-COVID times [37], in our study, no significant differences were found in hospitalization days or intra- and post-operative complications.

Medical oncology practices, in particular, chemotherapy and radiotherapy, were also affected by major changes during the COVID-19 pandemic, with the prioritization of curative over adjuvant therapies, as these intended to reduce local recurrence but not prolong survival [4,8,28,38]. A survey among the members of the Multicenter Italian Trials in Ovarian cancer and gynecologic malignancies (MITO) group between November 2020 and January 2021 revealed that 73% of physicians stopped chemotherapy or PARP-I treatment after a positive swab and resumed it when negative tests were confirmed [39]. Our data do not demonstrate significant modifications to oncological treatments, though a higher percentage of vulvar cancer patients were submitted to chemoradiotherapy in the pandemic recovery period (40.0%) and more patients were selected for palliative care in the pandemic with high restrictions period (12.5%). Furthermore, the median days between surgery and adjuvant therapy in ovarian cancer patients was also higher in the pandemic recovery period than in the other periods, which could be justified by the oncology services readjustment after the pandemic with high restrictions period in order to respond to all pending requests for all types of cancer and specialties, besides issues related to human resources.

A major strength of the present study is that it presents a continuous evaluation of the impact of the COVID-19 pandemic on the diagnosis and management of gynecological cancer over the pre-pandemic, pandemic with high restrictions and pandemic recovery periods instead of including only its initial repercussions as reported in the majority of the published evidence. In those cases, the impingement of the virus was completely new, none of the patients were vaccinated, and protective measures were yet undeveloped. Moreover, attending to the major impacts that this pandemic had on gynecologic cancer patients [40,41], our findings can have important implications on clinical practice and will also improve evidence for future pandemics and the planning of relevant studies during a worldwide crisis in the future.

Nonetheless, the conclusions from this study should be evaluated within the context of its potential limitations. First, its retrospective design may introduce inherent bias considering some important data that were not considered in this study, namely, other comorbidities like obesity, diabetes, asthma or inflammatory/autoimmune disorders that could impact the immunologic responses to SARS-CoV2 infection and could be of particular importance in cancer patient outcomes. In addition, the patients’ status of SARS-CoV2 infection was not analyzed, as this study aimed to assess the overall impact of the pandemic on patients with gynecological malignancies and was not limited to patients with proven infection. Other important limitations of our study include its small sample size and the derivation of data from a single institution, which makes it pertinent to carry out a multicenter study, including a larger and more diversified sample, with a prospective analysis of the impact of the COVID-19 pandemic on gynecologic cancer patient outcomes.

## 5. Conclusions

Collectively, our results indicate that the diagnosis and management of gynecological cancer were impacted by the COVID-19 pandemic. Regarding cancer diagnosis, a decreased number of new cases of cervical cancer occurred in the pandemic with high restrictions period, probably due to patients’ concerns about SARS-CoV2 infection, limited access to primary care services and the interruption of the cervical cancer screening program. Although some aggressive histological types of endometrial and ovarian cancer were more common in the pandemic recovery group, no differences were demonstrated concerning tumor staging in all gynecological cancers. Modifications to cancer management were also reported in most of the gynecological malignancies, namely, on time to first treatment, chosen therapeutic options and surgical approaches.

As these conclusions are associated with important clinical and healthcare implications, further studies, particularly with a prospective analysis and a larger study population, should be conducted to best address the survival rates and the actual medium- and long-term outcomes of patients with gynecological cancer.

## Figures and Tables

**Table 1:** Epidemiological, clinical and pathological patterns of patients diagnosed with endometrial cancer (*n* = 120).

	Pre-Pandemic (2019)	Pandemic with High Restrictions (2020)	Pandemic Recovery (2021)	*p*
**Number of patients, *n***	36	37	47	
**Age at diagnosis, median (IQR; Min-Max), years**	73.5 (13; 47–88)	66.0 (15; 35–86)	69.0 (15; 50–84)	0.046 *
**Postmenopausal status, *n* (%)**	35 (97.2)	34 (91.9)	46 (97.9)	0.349
**Histological type, *n* (%)** Endometrioid Serous Clear cells Undifferentiated Carcinosarcoma Mixed	17 (47.2) 15 (41.7) 0 (0.0) 1 (2.8) 2 (5.5) 1 (2.8)	30 (81.1) 2 (5.4) 3 (8.1) 1 (2.7) 0 (0.0) 1 (2.7)	34 (72.3) 3 (6.4) 1 (2.1) 3 (6.4) 2 (4.3) 4 (8.5)	0.001 *
**Tumor staging ^1^, *n* (%)** Initial stage (I-II) Advanced stage (III-IV)	23 (63.9) 13 (36.1)	32 (86.5) 5 (13.5)	38 (80.9) 9 (19.1)	0.054
**Time between first MDTM and treatment initiation, median (IQR; Min-Max), days**	23.0 (21; 6–55)	34.0 (22; 5–76)	36.0 (25; 13–147)	0.002 *
**Neoadjuvant therapy, *n* (%)**	0 (0.0)	1 (2.7)	0 (0.0)	0.323
**Surgical approach** Laparoscopy, *n* (%) Laparotomy, *n* (%) Laparotomy preceded by diagnostic laparoscopy, *n* (%)	9 (32.1) 18 (64.3) 1 (3.6)	5 (14.3) 25 (71.4) 5 (14.3)	16 (36.4) 216 (36.4) 12 (27.3)	0.006 *
**SLN biopsy, *n* (%)**	0 (0.0)	4 (11.1)	24 (54.5)	<0.001 *
**Hospitalization days, median (IQR; Min-Max)**	6.5 (4; 2–29)	6.0 (4; 2–41)	5.0 (4; 2–23)	0.158
**Intra- and post-operative complications ^2^, *n* (%)**	3 (10.7)	3 (11.5)	11 (25.6)	0.176
**Time between surgery and histological assessment, median (IQR; Min-Max), days**	28.0 (14; 8–44)	20.0 (11; 6–63)	16.0 (15; 7–42)	0.007 *
**Time between surgery and adjuvant therapy, median (IQR; Min-Max), days**	76.0 (20; 41–194)	63.5 (14; 35–103)	71 (29; 3–160)	0.314

Legend: IQR—interquartile range; MDTM—multidisciplinary team meeting; SLN—sentinel lymph node. ^1^ Tumor staging was surgical in most cases, considering the revised International Federation of Gynecology and Obstetrics (FIGO) Classification 2009; when surgery was contraindicated, clinical staging was assessed on the basis of FIGO Classification 1971 [19]. ^2^ Pre-pandemic (2019): infection and dehiscence of the surgical wound (*n* = 3); pandemic with high restrictions (2020): hemoperitoneum (*n* = 1), abdominal hematoma with aponeurotic dehiscence (*n* = 1), bladder injury (*n* = 1); pandemic recovery (2021): vesical dysfunction (*n* = 1), pelvic hematoma/abscess (*n* = 3), paralytic ileus (*n* = 1), ureter/bladder/vascular injury (*n* = 3), aponeurotic dehiscence (*n* = 2), pulmonary thromboembolism (*n* = 1). * Statistically significant differences for a significance level of 0.05.

**Table 2:** Epidemiological, clinical and pathological patterns of patients diagnosed with ovarian, fallopian tube and primary peritoneal cancer (*n* = 58).

	Pre-Pandemic (2019)	Pandemic with High Restrictions (2020)	Pandemic Recovery (2021)	*p*
**Number of patients, *n***	18	20	20	
**Age at diagnosis, median (IQR; Min-Max), years**	62.5 (18; 38–79)	63.5 (17; 31–84)	63 (27; 28–82)	0.759
**Postmenopausal status, *n* (%)**	14 (77.8)	17 (85.0)	15 (75.0)	0.724
**Histological type, *n* (%)** Serous Endometrioid Mucinous Clear cells Undifferentiated Carcinosarcoma Mixed Borderline Malignant non-epithelial tumors Unknown	8 (44.4) 1 (5.6) 2 (11.1) 0 (0.0) 3 (16.7) 0 (0.0) 1 (5.6) 0 (0.0) 2 (11.1) 1 (5.6)	12 (60.0) 0 (0.0) 0 (0.0) 1 (5.0) 0 (0.0) 0 (0.0) 1 (5.0) 3 (15.0) 0 (0.0) 3 (15.0)	4 (20.0) 0 (0.0) 0 (0.0) 2 (10.0) 1 (5.0) 1 (5.0) 0 (0.0) 9 (45.0) 2 (10.0) 1 (5.0)	0.018 *
**Tumor staging ^1^, *n* (%)** Initial stage (I-II) Advanced stage (III-IV) Unknown	7 (38.9) 10 (55.6) 1 (5.6)	11 (55.0) 9 (45.0) 0 (0.0)	14 (70.0) 6 (30.1) 0 (0.0)	0.254
**Time between first MDTM and treatment initiation, median (IQR; Min-Max), days**	34.0 (23; 1–49)	22.0 (22; 1–104)	36.0 (29; 5–215)	0.293
**Neoadjuvant therapy, *n* (%)**	6 (33.3)	11 (55.0)	2 (10.0)	0.010 *
**Diagnostic/staging surgery** Laparoscopy, *n* (%) Laparotomy, *n* (%)	4 (66.7) 2 (33.3)	7 (100.0) 0 (0.0)	6 (66.7) 3 (33.3)	0.221
**Primary cytoreductive surgery** Laparoscopy, *n* (%) Laparotomy, *n* (%) Laparotomy preceded by diagnostic laparoscopy, *n* (%)	0 (0.0) 4 (50.0) 4 (50.0)	1 (11.1) 5 (55.6) 3 (33.3)	0 (0.0) 10 (100.0) 0 (0.0)	0.073
**Interval debulking surgery** Laparotomy, *n* (%) Laparotomy preceded by diagnostic laparoscopy, *n* (%)	2 (100.0) 0 (0.0)	3 (37.5) 5 (62.5)	0 (0.0) 1 (100.0)	0.179
**Hospitalization days ^2^, median (IQR; Min-Max)**	8.0 (9; 1–21)	6.0 (5; 2–21)	5.5 (4; 2–22)	0.143
**Intra- and post-operative complications ^3^, *n* (%)**	3 (21.4)	2 (11.8)	3 (16.7)	0.768
**Time between surgery and histological assessment, median (IQR; Min-Max), days**	15.0 (29; 1–45)	13.5 (8; 6–31)	11.0 (11; 3–51)	0.693
**Time between surgery and adjuvant therapy, median (IQR; Min-Max), days**	49.5 (29; 21–63)	42.0 (31; 29–71)	79.0 (37; 64–108)	0.016 *

Legend: IQR—interquartile range; MDTM—multidisciplinary team meeting. ^1^ Tumor staging was assessed using the International Federation of Gynecology and Obstetrics (FIGO) Classification 2014 [20]. ^2^ Hospitalization days refer to the first surgical intervention. ^3^ Intra- and post-operative complications were considered for all surgical procedures; pre-pandemic (2019): infection of the surgical wound (*n* = 1), paralytic ileus (*n* = 1), thrombophlebitis (*n* = 1); pandemic with high restrictions (2020): infection of the surgical wound (*n* = 1), bowel injury and peritonitis (*n* = 1); pandemic recovery (2021): postoperative infection (*n* = 1), hemoperitoneum (*n* = 1), ureter injury (*n* = 1). * Statistically significant differences for a significance level of 0.05.

**Table 3:** Epidemiological, clinical and pathological patterns of patients diagnosed with cervical cancer (*n* = 24).

	Pre-Pandemic (2019)	Pandemic with High Restrictions (2020)	Pandemic Recovery (2021)	*p*
**Number of patients, *n***	8	6	10	
**Age at diagnosis, median (IQR; Min-Max), years**	63.5 (27; 35–77)	69.0 (23; 55–84)	47.5 (32; 39–80)	0.251
**Postmenopausal status, *n* (%)**	6 (75.0)	6 (100.0)	4 (40.0)	0.040 *
**Histological type, *n* (%)** Squamous cell carcinoma Adenocarcinoma Carcinosarcoma/sarcoma	6 (75.0) 1 (12.5) 1 (12.5)	4 (66.7) 2 (33.3) 0 (0.0)	7 (70.0) 2 (20.0) 1 (10.0)	0.835
**Tumor staging**^1^**, *n* (%)** Initial stage (IA1-IB2, IIA1) Advanced stage (IB3, IIA2, IIB-IV)	1 (12.5) 7 (87.5)	1 (16.7) 5 (83.3)	3 (30.0) 7 (70.0)	0.635
**Time between first MDTM and treatment initiation, median (IQR; Min-Max), days**	52.0 (43; 35–90)	60.0 (-; 20–123)	41.0 (26; 20–71)	0.445
**Therapeutic management, *n* (%)** Chemoradiotherapy Surgery Palliative No treatment ^2^	0 (0.0) 3 (37.5) 2 (25.0) 3 (37.5)	2 (33.3) 1 (16.7) 0 (0.0) 3 (50.0)	1 (40.0) 3 (30.0) 2 (20.0) 1 (10.0)	0.292
**Hospitalization days, median (Min-Max)**	5.0 (5–8)	-	7.0 (6–8)	0.660
**Intra- and post-operative complications, *n* (%)**	0 (0.0)	0 (0.0)	0 (0.0)	-
**Time between surgery and histological assessment, median (Min-Max), days**	17.0 (13–32)	-	27.0 (21–48)	0.319
**Adjuvant therapy, *n* (%)**	1 (12.5)	0 (0.0)	1 (10.0)	0.683

Legend: IQR—interquartile range; MDTM—multidisciplinary team meeting. ^1^ Tumor staging was assessed using the International Federation of Gynecology and Obstetrics (FIGO) Classification 2018 [21]. ^2^ Patients under surveillance, lost to follow-up or death. * Statistically significant differences for a significance level of 0.05.

**Table 4:** Epidemiological, clinical and pathological patterns of patients diagnosed with vaginal and vulvar cancer (*n* = 21).

	Pre-Pandemic (2019)	Pandemic with High Restrictions (2020)	Pandemic Recovery (2021)	*p*
**Number of patients, *n***	8	8	5	
**Age at diagnosis, median (IQR; Min-Max), years**	71.0 (21; 54–86)	80.0 (17; 72–96)	68.0 (42; 32–89)	0.095
**Postmenopausal status, *n* (%)**	8 (100.0)	8 (100.0)	3 (60.0)	0.029 *
**Histological type, *n* (%)** Squamous cell carcinoma Melanoma Paget´s disease of the vulva Basal cell carcinoma	4 (50.0) 1 (12.5) 1 (12.5) 2 (25.0)	7 (87.5) 0 (0.0) 0 (0.0) 1 (12.5)	5 (100.0) 0 (0.0) 0 (0.0) 0 (0.0)	0.440
**Tumor staging**^1^**, *n* (%)** Initial stage (IA, IB) Advanced stage (II-IV) Unknown	5 (62.5) 0 (0.0) 3 (37.5)	5 (62.5) 3 (37.5) 0 (0.0)	2 (40.0) 3 (60.0) 0 (0.0)	0.051
**Time between first MDTM and treatment initiation, median (IQR; Min-Max), days**	27.0 (-; 27–74)	27.0 (23; 13–43)	49.0 (73; 21–131)	0.354
**Therapeutic management, *n* (%)** Chemoradiotherapy Surgery ^2^ Palliative No treatment ^3^	0 (0.0) 3 (37.5) 0 (0.0) 5 (62.5)	0 (0.0) 5 (62.5) 1 (12.5) 2 (25.0)	2 (40.0) 3 (60.0) 0 (0.0) 0 (0.0)	0.053
**Hospitalization days, median (Min-Max)**	21.0 (-; 3–44)	30.0 (37; 3–45)	9.0 (-; 3–12)	0.448
**Intra- and post-operative complications ^4^, *n* (%)**	1 (33.3)	4 (80.0)	1 (33.3)	0.302
**Time between surgery and histological assessment, median (Min-Max), days**	50.0 (-; 16–90)	30.0 (26; 12–42)	31.0 (-; 25–35)	0.517

Legend: IQR—interquartile range; MDTM—multidisciplinary team meeting. ^1^ Tumor staging was assessed using the revised International Federation of Gynecology and Obstetrics (FIGO) Classification 2009 [19] and TNM Classification of Malignant Tumors (TNM) 2017 [22]. ^2^ In two cases, patients were submitted to neoadjuvant chemoradiotherapy. ^3^ Patients under surveillance, lost to follow-up or death. ^4^ Pre-pandemic (2019): infection and dehiscence of the surgical wound (*n* = 1); pandemic with high restrictions (2020): vulvar hemorrhage (*n* = 1), infection and dehiscence of the surgical wound (*n* = 3); pandemic recovery (2021): infection and dehiscence of the surgical wound (*n* = 1). * Statistically significant differences for a significance level of 0.05.

## Data Availability

The data presented in this study are available on request from the corresponding author.
